# Reading Conflicting Socio-Scientific Issue Texts: Visual and Post-Reading Myside Bias

**DOI:** 10.3390/jemr19050118

**Published:** 2026-10-13

**Authors:** Shuo Ban, Siti Nur Diyana Mahmud, Kamisah Osman

**Affiliations:** STEM Enculturation Research Centre, Faculty of Education, Universiti Kebangsaan Malaysia, Bangi 43600, Malaysia

**Keywords:** epistemic beliefs, prior knowledge, eye tracking, myside bias, socio-scientific issues

## Abstract

This study examined whether university students display myside bias during and after reading conflicting socio-scientific issue (SSI) texts about genetically modified foods. Fifty-seven students read supporting and opposing claims while wearing eye-tracking glasses. Visual myside bias, defined as greater visual attention to information consistent with readers’ pre-reading stance than to information inconsistent with that stance, was assessed using percent time spent (PTS) and revisited fixation count (RFC) in myside and otherside areas of interest. Prior knowledge, scientific epistemic beliefs, epistemic emotions, and a post-reading argument generation task were also assessed. Students devoted significantly more visual attention to myside information on both PTS and RFC, showing that myside bias emerged during reading as well as in post-reading argument generation. Prior knowledge and scientific epistemic beliefs negatively predicted both visual and post-reading bias. Negative epistemic emotions were positively correlated with both bias measures but were not significant after cognitive variables were controlled. Longer viewing time and more frequent revisits did not necessarily indicate higher-quality informal reasoning; when directed toward myside information, they reflected stance-consistent processing. These findings provide process-based evidence of biased SSI reading and highlight knowledge and epistemic beliefs as potential supports for more balanced, evidence-oriented engagement with conflicting information.

## 1. Introduction

Socio-scientific issues (SSI), such as genetically modified (GM) foods, nuclear energy, global warming, and vaccination, often involve conflicting scientific claims, uncertainties, and competing social perspectives [1]. As students encounter information about these issues through educational materials and online media, they need to consider evidence supporting different positions. However, their engagement with conflicting information may be influenced by their existing views. Rather than attending to and evaluating information from both sides in a balanced manner, students may preferentially process information that supports their own positions. This tendency, known as myside bias, can be reflected in how individuals select, interpret, evaluate, and generate arguments about controversial issues [2,3,4,5,6,7,8].

Previous research has commonly examined myside bias through post-reading measures, including argument generation and informal reasoning tasks [9,10]. These measures provide evidence of how individuals express and organize arguments after encountering conflicting information, but they offer limited insight into how information is attended to during reading. Myside bias may emerge before readers construct their final responses, through differences in the attention they allocate to information consistent or inconsistent with their initial positions. Examining visual attention during reading can therefore complement post-reading measures and provide a more detailed account of position-consistent processing.

Eye-tracking technology offers a means of examining how readers distribute visual attention across conflicting information [11,12,13]. Previous eye-tracking research has investigated attention allocation during the reading of controversial issues and its relationship with subsequent reasoning outcomes [7,14]. Nevertheless, further research is needed to examine how differences in attention to myside and otherside information are associated with myside bias in post-reading argument generation. In the present study, visual myside bias refers to the relative allocation of visual attention to information consistent with a reader’s pre-reading stance compared with information inconsistent with that stance. This bias is examined using percent time spent (PTS) and revisited fixation count (RFC) in myside and otherside areas of interest. These measures capture different aspects of visual engagement, but neither longer viewing time nor more frequent revisits, on its own, establishes whether information has been critically evaluated or accepted. Accordingly, examining the direction of visual attention alongside post-reading argument generation may provide a more informative account of myside bias than either measure alone.

Individual cognitive and affective characteristics may also be associated with how students process conflicting SSI information [15]. Prior knowledge may support the comprehension and comparison of competing claims [16,17,18,19], while scientific epistemic beliefs may be related to how students approach uncertainty, evidence, and differing explanations [20,21,22]. Epistemic emotions experienced during reading may likewise accompany differences in engagement with information that supports or challenges an existing position [23,24,25]. Although these factors have been examined in relation to controversial information processing, their associations with visual myside bias during reading and myside bias in subsequent argument generation require further investigation within the same SSI reading context [26].

The present study used conflicting texts about the safety of GM foods to examine visual attention during reading and myside bias in post-reading argument generation among college students. Participants’ positions were measured before reading and used to classify the supporting and opposing information as myside or otherside for each individual. By combining eye-tracking measures with a post-reading argument generation task, the study examined whether participants allocated more visual attention to myside information, whether visual and post-reading myside bias were associated, and how prior knowledge, scientific epistemic beliefs, and epistemic emotions were related to these two forms of bias. The study addressed the following research questions:

1. Do college students allocate different amounts of visual attention to myside and otherside information when reading conflicting SSI texts?
2. How is visual myside bias during reading associated with post-reading myside bias in argument generation?
3. How are prior knowledge, scientific epistemic beliefs, and epistemic emotions associated with visual myside bias during SSI reading?
4. How are prior knowledge, scientific epistemic beliefs, and epistemic emotions associated with post-reading myside bias in argument generation?

### 1.1. Myside Bias in SSI Reading

Myside bias refers to the tendency to process information, evaluate evidence, or generate arguments in ways that favor one’s existing position [9,27,28]. Although it is closely related to confirmation bias, myside bias specifically emphasizes the role of an individual’s own stance in reasoning about an issue. It also differs from belief bias, which concerns the influence of prior beliefs about the plausibility of a conclusion on reasoning and judgment [29,30]. Myside bias is particularly relevant when individuals encounter controversial issues involving competing claims and perspectives, because their existing positions may shape how they engage with information that supports or challenges their views [10,28].

Socio-scientific issues (SSI) provide an important context for examining myside bias. These issues involve scientific knowledge and evidence alongside uncertainty, ethical considerations, social interests, and competing values [31,32,33,34,35]. When reading conflicting SSI information, students may encounter claims that are consistent with their initial positions as well as claims that challenge those positions. Their responses may therefore reflect both the evaluation of scientific evidence and the influence of their existing views. In this context, myside bias may be expressed through preferential attention to stance-consistent information during reading or through the generation of more arguments supporting one’s initial position afterward. Such patterns are relevant to understanding how students engage with competing evidence when reasoning about SSI [7,36,37].

Previous research has examined myside bias through argument evaluation, informal reasoning, and written argumentation tasks [9,10,38,39,40]. These approaches provide information about how individuals evaluate or construct arguments, but post-reading responses alone do not fully reveal how attention was allocated while the conflicting information was being read. Eye-tracking research on controversial SSI reading has shown that visual attention to information supporting or challenging a reader’s position can be examined alongside subsequent reasoning outcomes [7]. Investigating visual attention and post-reading argument generation in the same participants may therefore clarify whether stance-consistent patterns observed during reading are associated with those expressed in subsequent argumentation.

The present study examined myside bias in the context of conflicting information about the safety of genetically modified (GM) foods, a topic involving scientific claims and societal concerns [41,42]. Participants’ positions on GM food safety were measured before reading and used to classify the supporting and opposing passages as myside or otherside information for each participant. Visual myside bias was operationalized as the difference in visual attention allocated to these two types of information, whereas post-reading myside bias was operationalized as the difference between the numbers of myside and otherside arguments generated after reading. These measures capture different aspects of stance-consistent processing: the former concerns attention allocation during reading, while the latter concerns the balance of arguments generated afterward. Examining their association allows the study to investigate how visual attention patterns during SSI reading relate to subsequent argument generation, without assuming that attention allocation directly causes post-reading bias.

### 1.2. Visual Myside Bias During SSI Reading

Myside bias has commonly been examined through argument evaluation, informal reasoning, and written argumentation tasks [9,10,39,40]. These approaches reveal how individuals evaluate or generate arguments, but they provide limited information about the allocation of attention while conflicting information is being read. Visual attention is relevant to this process because readers may devote different amounts of time to information that supports or challenges their initial positions. Eye-tracking studies of controversial texts have demonstrated that readers’ existing beliefs and positions are associated with differences in how they attend to conflicting information [7,21,43]. Examining these differences during reading can therefore complement post-reading measures of myside bias.

Eye tracking provides a continuous record of where readers direct their gaze and how long they attend to particular regions of a text. In studies involving conflicting information, eye-movement measures have been used to investigate attention allocation, the processing of competing claims, and readers’ engagement with information from different sources [21,43,44,45]. However, eye movements do not directly reveal whether readers accept a claim, evaluate its evidential quality, or integrate it into their understanding. Greater visual attention may reflect several processes, including sustained engagement, comprehension difficulty, or repeated examination of information. Consequently, the interpretation of eye-tracking measures depends on the reading task and the information to which attention is directed.

The present study used two eye-tracking measures to examine visual myside bias: percent time spent (PTS) and revisited fixation count (RFC). PTS represents the proportion of valid viewing time allocated to a specified area of interest (AOI), whereas RFC represents the number of returns to an AOI after the reader’s gaze has left it. PTS therefore captures the relative duration of visual attention, while RFC captures the frequency with which readers return to previously viewed information. When the AOIs are classified according to a reader’s pre-reading stance, differences in these measures can indicate whether relatively more viewing time and more frequent returns are directed toward myside or otherside information. Nevertheless, higher PTS or RFC in a particular AOI does not, by itself, establish the purpose or quality of the processing that occurred there.

Previous research provides a basis for examining visual attention alongside subsequent reasoning about controversial issues. In an eye-tracking study of online reading about nuclear energy, Yen and Wu [7] investigated the relationships between students’ engagement with conflicting information and their informal reasoning outcomes. Research on controversial historical accounts has also examined myside bias during reading and in subsequent written responses [39]. Building on these approaches, the present study examines whether visual attention differences between myside and otherside information are associated with the balance of arguments generated after reading an SSI text about genetically modified foods. Visual myside bias is operationalized as the myside–otherside difference in PTS and RFC, while post-reading myside bias is measured by the difference between the numbers of arguments supporting and opposing each participant’s initial position. Examining the associations between these measures allows the study to investigate whether stance-consistent attention patterns during reading correspond to stance-consistent argument generation afterward, without assuming that visual attention directly causes post-reading bias.

### 1.3. Cognitive and Affective Predictors of Myside Bias in SSI Reading

Myside bias in SSI reading may be associated not only with students’ initial positions but also with individual differences in their knowledge, epistemic beliefs, and emotional experiences. Reading conflicting SSI information requires students to understand competing claims, consider the evidence presented by different sources, and respond to uncertainty. These processes may vary according to students’ cognitive and affective characteristics. Previous research on controversial historical texts has examined the relationships of epistemic beliefs, emotions, and prior knowledge with myside bias during reading and in subsequent writing [39]. However, further research is needed to examine how these factors are associated with both visual attention allocation and post-reading argument generation within the same SSI reading task.

Prior knowledge may be relevant to how students understand and compare conflicting information. Students with greater topic knowledge may be better able to identify the scientific concepts, evidence, and limitations underlying competing claims, whereas students with less knowledge may experience greater difficulty interpreting unfamiliar information [16,17,18]. In SSI reading, prior knowledge may therefore be associated with the extent to which students attend to information supporting or challenging their initial positions. However, greater knowledge does not necessarily ensure balanced information processing, because readers may also use what they know to support an existing position [9,21]. Examining the relationship between prior knowledge and myside bias can help clarify whether students’ knowledge of a particular SSI topic is associated with their attention allocation during reading and the balance of arguments they generate afterward.

Scientific epistemic beliefs concern individuals’ beliefs about scientific knowledge and how scientific claims are developed and justified. These beliefs include views about the source and certainty of knowledge, the development of scientific understanding, and the role of evidence in justifying scientific claims [20,21,22]. When students encounter conflicting SSI information, their epistemic beliefs may be associated with how they approach uncertainty, evaluate competing explanations, and consider evidence that challenges their existing positions. Previous studies have reported relationships between epistemic beliefs and the evaluation or processing of controversial information [39,46,47,48,49]. Students who recognize that scientific explanations can develop and that claims require evidential justification may be more willing to consider competing perspectives. Nevertheless, the relationship between epistemic beliefs and myside bias should be examined empirically rather than assuming that particular beliefs necessarily produce balanced reading. In the present study, scientific epistemic beliefs were examined in relation to both visual myside bias and post-reading argument generation.

Epistemic emotions are emotional experiences associated with activities involving knowledge and knowing, including encountering unfamiliar information, uncertainty, or conflicting explanations [23,24,25]. Reading competing claims about an SSI may elicit positive emotions, such as curiosity and interest, as well as negative emotions, such as anxiety, confusion, and frustration. These emotions may accompany differences in how students engage with conflicting information. For example, curiosity may be associated with further exploration, while anxiety or frustration may accompany difficulty processing information that challenges an existing position [23,25]. However, the implications of epistemic emotions are not necessarily uniform: confusion may also prompt further examination of evidence rather than avoidance. Previous research has examined epistemic emotions in relation to students’ thinking about SSI and their responses to information about genetically modified foods [17,50]. The present study therefore examined positive and negative epistemic emotions separately to investigate their associations with visual attention allocation and post-reading myside bias.

Taken together, prior knowledge, scientific epistemic beliefs, and epistemic emotions may be associated with different aspects of students’ engagement with conflicting SSI information. Examining these variables alongside eye-tracking measures and a post-reading argument generation task allows the present study to assess whether their associations with stance-consistent attention during reading correspond to their associations with subsequent argument generation. Because the study used an observational design, these relationships are interpreted as associations rather than evidence that the cognitive or affective variables directly cause myside bias.

## 2. Materials and Methods

### 2.1. Participants

Fifty-nine college students from Jining Polytechnic in China voluntarily participated in this study. The participants were enrolled in various majors, including engineering, tourism, psychology, education, and management. Two participants were excluded from the eye-tracking analysis because their proportions of valid gaze samples were below the predefined threshold of 90%. The final sample comprised 57 participants (25 males and 32 females), with a mean age of 20.3 years (SD = 2.34, range = 18–26 years). All participants were native Chinese speakers and were at least 18 years old.

Participants were recruited through campus recruitment announcements. All participants provided written informed consent before the experiment. Participation was voluntary, and each participant received a small gift (cost 5 USD) as a token of appreciation for their participation.

### 2.2. Materials

The stimulus was adapted from Chinese-language popular science and debate materials concerning GM food safety that were available in a university teaching material database. It was not translated directly from a single academic publication. The supporting passage drew in part on published research concerning long-term feeding of GM soybeans, whereas the opposing passage included claims that had circulated in public debate about GM crops. The researchers edited the two passages for clarity and to make their overall lengths as comparable as possible while preserving the intended meaning of the original materials. The Chinese text was presented on a single page and comprised three sections: Definition, Supporting Views, and Opposing Views (Figure 1). The Definition section introduced the basic concepts of genetic modification and GM foods. The Supporting Views section presented arguments supporting the safety and use of GM foods, whereas the Opposing Views section presented arguments questioning their safety and use. The reading material was obtained from the university database of popular science materials on genetically modified foods. The researchers adapted the two passages appropriately to make their lengths as similar as possible while preserving their scientific meaning and ensuring clarity of expression. An English translation of the stimulus is provided in Appendix A.

The two argumentative passages were closely matched in overall text length. Excluding punctuation, numerals, and other symbols, the Supporting Views section contained approximately 337 Chinese characters, whereas the Opposing Views section contained approximately 331 Chinese characters. The Supporting Views section contained 27 punctuation marks, compared with 25 in the Opposing Views section. Both passages presented a position on GM foods, supporting reasons, illustrative research claims, and a concluding statement. However, the Supporting Views section contained nine sentences, whereas the Opposing Views section contained six sentences. Thus, although the passages were comparable in overall length and argumentative structure, they were not identical in sentence length or linguistic complexity.

As shown in Figure 1, the Supporting Views section was presented in the left panel (AOI 1), whereas the Opposing Views section was presented in the right panel (AOI 2). Both panels occupied equivalent rectangular areas, each measuring 1070 × 755 pixels in the snapshot coordinate system. The spatial arrangement was identical for all participants, and the positions of the supporting and opposing passages were not counterbalanced.

The introductory Definition section was located above the two argumentative panels and was not included in either AOI. Only AOI 1 and AOI 2 were used in the myside–otherside comparisons. Accordingly, visual attention directed toward the introductory section or locations outside the two argumentative AOIs was not included in these comparisons. The supporting and opposing panels were subsequently classified as myside or otherside according to each participant’s pre-reading stance.

### 2.3. Instruments

This study used a prior knowledge test, a scientific epistemic beliefs questionnaire, an epistemic emotions questionnaire, an initial stance measure, and a post-reading argument generation task. The initial stance measure was administered before the reading task, whereas the epistemic emotions questionnaire and argument generation task were administered after reading. The instruments are described below.

#### 2.3.1. Prior Knowledge Test

The prior knowledge test was used to assess participants’ knowledge of genetically modified (GM) foods. Adapted from Muis et al. [17], the test consisted of 10 multiple-choice items covering knowledge related to GM foods (Appendix B). Each correct answer was scored as 1 point, and each incorrect answer was scored as 0 points, yielding a total score ranging from 0 to 10. Higher scores indicated greater prior knowledge of GM foods. In the present study, the 10-item prior knowledge test yielded a KR-20 coefficient of 0.603, indicating limited internal consistency. Item difficulty indices, expressed as the proportion of participants answering each item correctly, ranged from 0.035 to 1.000. Items 1–3 were answered correctly by all participants and therefore showed no score variance. Among the remaining items, corrected item–total correlations ranged from −0.157 to 0.610. Items 4 and 10 showed negative corrected item–total correlations (−0.112 and −0.157, respectively), whereas Items 5–9 showed positive corrected item–total correlations ranging from 0.365 to 0.610. Items 1–3 contributed no between-participant variance, and Items 4 and 10 contributed little discriminating information. Accordingly, the effective discriminating length of the prior knowledge measure in this sample was five items (Items 5–9). Full item-level statistics are reported in Appendix F.

#### 2.3.2. Scientific Epistemic Beliefs Questionnaire

The scientific epistemic beliefs questionnaire was adapted from Conley et al. [22] to assess participants’ beliefs about scientific knowledge and the process of scientific knowing (Appendix C). The questionnaire comprised 26 items across four dimensions: Source (5 items), Certainty (6 items), Development (6 items), and Justification (9 items). Participants rated each item on a 5-point Likert scale ranging from 1 (strongly disagree) to 5 (strongly agree).

Items in the Source and Certainty dimensions were reverse-scored before the dimension scores were calculated because agreement with these items reflected less sophisticated scientific epistemic beliefs. Items in the Development and Justification dimensions retained their original scoring direction. A mean score was calculated separately for each dimension, with higher scores indicating more sophisticated scientific epistemic beliefs. The overall scientific epistemic beliefs score used in the analyses was calculated by averaging the four dimension scores with equal weighting.

In the present study, Cronbach’s α values for the Source, Certainty, Development, and Justification dimensions of the Chinese version were 0.69, 0.76, 0.82, and 0.77, respectively. The reliability coefficient for the overall composite score was 0.554.

#### 2.3.3. Epistemic Emotions Questionnaire

The epistemic emotions questionnaire was adapted from Heddy et al. [50] to assess participants’ emotional experiences during the reading of the conflicting SSI text on GM foods (Appendix D). The questionnaire included six positive epistemic emotions and seven negative epistemic emotions. After completing the reading task, participants retrospectively rated the intensity of each emotion they had experienced during reading on a 5-point Likert scale ranging from 1 (not at all) to 5 (very strongly).

Positive and negative epistemic emotions were scored separately by summing the corresponding item scores. The possible total scores ranged from 6 to 30 for positive epistemic emotions and from 7 to 35 for negative epistemic emotions. Higher scores indicated greater reported intensity of the corresponding category of epistemic emotions.

In the present study, Cronbach’s α was 0.927 for the positive epistemic emotions subscale and 0.856 for the negative epistemic emotions subscale.

#### 2.3.4. Initial Stance Measure and Post-Reading Argument Generation Task

Participants’ initial stance toward the safety of GM foods was measured before they read the conflicting text. They were presented with the statement “Genetically modified foods are safe” and asked to indicate their agreement on a 6-point scale ranging from 1 (strongly disagree) to 6 (strongly agree). Responses of 1, 2, or 3 were classified as an initial disagreeing stance, whereas responses of 4, 5, or 6 were classified as an initial agreeing stance. The original six-point responses were retained for the supplementary analysis of stance extremity.

Each participant’s pre-reading stance was used to define myside and otherside information throughout the study. For participants who initially agreed with the statement, information supporting the safety of GM foods was classified as myside information, whereas information opposing the statement was classified as otherside information. For participants who initially disagreed, the reverse classification was applied. No second attitude measurement was administered after reading.

Post-reading myside bias was assessed using an argument generation task adapted from previous research on informal reasoning and myside bias [7,51,52]. After reading the conflicting text, participants completed a numerical sequence distractor task consisting of six problems unrelated to GM foods. Each problem could be advanced manually after the participant responded; otherwise, the program automatically advanced to the next problem after a maximum of 30 s. Participants were then presented again with the statement “Genetically modified foods are safe” and proceeded directly to the argument generation task, in which they were asked to generate as many arguments as possible that supported their initial position and as many arguments as possible that opposed it.

The generated arguments were first classified as supporting, opposing, or other, as described in Section 2.6. Arguments classified as supporting or opposing were then recoded as myside or otherside arguments according to each participant’s pre-reading stance. The post-reading myside bias index was calculated as follows:

Post-reading myside bias index = N _myside arguments_ − N _otherside arguments_

A higher positive score indicated that a participant generated more arguments consistent with their initial stance than arguments inconsistent with that stance. A score of zero indicated equal numbers of myside and otherside arguments, whereas a negative score indicated that more otherside than myside arguments were generated.

### 2.4. Apparatus

Eye movements were recorded using Tobii Pro Glasses 2 (Tobii Pro, Stockholm, Sweden) at a sampling frequency of 50 Hz. The reading material was presented on a 15-inch Dell Precision 5570 (Dell Inc., Round Rock, TX, USA) laptop with a screen resolution of 1920 × 1200 pixels. Participants wore the eye-tracker while seated in front of the screen. Before recording, each participant completed the standard one-point calibration procedure provided by Tobii Pro Glasses 2. A separate numerical calibration accuracy value was not retained during data collection.

Eye-tracking data were recorded using Tobii Pro Glasses Controller and subsequently processed in Tobii Pro Lab. Fixations were identified using the Tobii I-VT (Attention) gaze filter. Noise reduction was performed using a moving median filter with a window size of three samples. Gaze velocity was calculated using a 20-ms window, and the velocity threshold for fixation classification was set to 100°/s. Adjacent fixations were merged when the interval between them did not exceed 75 ms and their angular separation did not exceed 0.5°. Fixations shorter than 60 ms were discarded. Gap-fill interpolation was disabled.

Gaze data from the scene camera recordings were mapped onto a static snapshot of the reading stimulus using the automatic snapshot mapping function in Tobii Pro Lab. The supporting and opposing text panels were defined as fixed areas of interest (AOIs) in the snapshot coordinate system, and the mapped gaze data were used to extract the corresponding AOI-based eye-tracking measures. The AOI dimensions reported in Section 2.2 refer to coordinates on this static snapshot and are therefore distinct from the laptop display resolution of 1920 × 1200 pixels. A separate numerical estimate of mapping error was not retained.

The proportion of valid gaze samples was used to assess recording quality. Recordings with fewer than 90% valid gaze samples were excluded from the eye-tracking analysis.

### 2.5. Procedure

The experiment was conducted in a quiet, enclosed laboratory at Jining Polytechnic. Before the experiment, participants were informed about the study procedures and provided written informed consent. Each participant completed the experimental session individually under the supervision of researchers.

First, participants completed the prior knowledge test on genetically modified (GM) foods and the scientific epistemic beliefs questionnaire. This stage took approximately 10 min. Before reading the conflicting text, participants indicated their initial stance toward the statement “Genetically modified foods are safe” using the 6-point scale described in Section 2.3.4. This pre-reading response was recorded and subsequently used to classify the supporting and opposing information as myside or otherside for each participant. The sequence of the experimental procedures reported here was verified against the original executable experimental program used for data collection. The program enforced the task order and recorded participants’ responses during the session. The original experimental program has been provided to the Editorial Office as documentary evidence of the procedure.

Participants then wore the Tobii Pro Glasses 2 eye-tracker and completed the standard one-point calibration procedure. After calibration, they sat in front of the laptop screen and pressed the space bar when they were ready to begin the reading task. A black fixation dot appeared in the center of the screen for 2000 ms, after which the conflicting text on GM foods was automatically presented. Participants read the text at their own pace. When they had finished reading, they pressed the space bar again to end the reading task and subsequently removed the eye-tracker.

Following the reading task, participants completed the epistemic emotions questionnaire to retrospectively report the emotions they had experienced while reading. They then completed a numerical sequence distractor task consisting of six problems unrelated to GM foods. Participants could manually advance to the next problem after responding. If they did not advance manually, the program automatically proceeded to the next problem after 30 s. No immediate feedback or explanation was provided.

Immediately after the distractor task, participants completed the post-reading argument generation task. They were presented again with the statement “Genetically modified foods are safe” and asked to generate as many arguments as possible that supported their initial position and as many arguments as possible that opposed it. The statement was presented to prompt argument generation; participants were not asked to rate their attitude again after reading.

After completing all experimental tasks, participants were debriefed about the nature of the stimulus materials. They were informed that the passages had been adapted for experimental purposes from materials used in public and educational discussions of GM food safety, that some claims represented contested positions in public debate, and that inclusion of those claims did not imply endorsement of their scientific validity by the researchers.

The entire experimental session lasted approximately 40 min. The experimental procedure is illustrated in Figure 2.

### 2.6. Argument Coding

To calculate post-reading myside bias, the arguments generated by the 57 participants were coded into three primary categories: supporting, opposing, and other. A total of 322 responses were generated during the argument generation task. Supporting arguments referred to statements supporting the claim that genetically modified foods are safe, whereas opposing arguments referred to statements challenging this claim. The “other” category included vague or irrelevant statements, arguments without a clear position, illogical responses, and repeated arguments.

Two researchers independently classified all 322 responses into three primary categories: supporting, opposing, and other. Their initial classifications agreed on 319 responses and differed on three, yielding an initial percentage agreement of 99.1%. The three disagreements were resolved through discussion, and a consensus classification was reached for each response. The final consensus codes were used in the subsequent analyses.

Following the consensus coding procedure, 317 responses were classified as supporting or opposing arguments and were retained for the myside bias analysis. The remaining five responses (1.6%) were classified as other and excluded from the calculation of the myside bias index.

The supporting and opposing arguments were subsequently recoded as myside or otherside according to each participant’s pre-reading stance. For participants who initially agreed that genetically modified foods are safe, supporting arguments were classified as myside arguments and opposing arguments as otherside arguments. For participants who initially disagreed with the statement, opposing arguments were classified as myside arguments and supporting arguments as otherside arguments. This second-stage classification was rule-based and did not involve an additional subjective coding decision.

The final dataset contained 189 myside arguments and 128 otherside arguments. For each participant, the post-reading myside bias index was calculated by subtracting the number of otherside arguments from the number of myside arguments, as described in Section 2.3.4. Responses classified as other were not included in this calculation.

### 2.7. Eye-Tracking Measures

The eye-tracking measures used in this study were percent time spent (PTS) and revisited fixation count (RFC). Both measures were extracted from the gaze data mapped onto the static snapshot of the reading stimulus in Tobii Pro Lab. The supporting and opposing text panels were defined as two separate areas of interest (AOIs), as described in Section 2.2 [53].

PTS was used to assess the proportion of viewing time allocated to each argumentative AOI. It was calculated as the percentage of valid viewing time spent within the corresponding AOI. Higher PTS values indicated a greater proportion of viewing time allocated to that area. Because the introductory definition section and locations outside the two argumentative AOIs were not included in the myside–otherside comparison, the PTS values for the supporting and opposing panels were not expected to sum to 100%.

RFC was operationally defined as the number of times a participant returned to an AOI after their gaze had previously left that AOI. The initial entry into an AOI was not counted as a revisit. Each subsequent return to the AOI was counted as one revisit, regardless of the number of fixations occurring during that visit. Higher RFC values indicated more frequent returns to the corresponding argumentative AOI during reading.

The supporting and opposing AOIs were subsequently classified as myside or otherside according to each participant’s pre-reading stance toward the statement “Genetically modified foods are safe”. For participants who initially agreed with the statement, the supporting panel (AOI 1, left) was classified as the myside AOI, whereas the opposing panel (AOI 2, right) was classified as the otherside AOI. For participants who initially disagreed, the opposing panel was classified as the myside AOI, and the supporting panel was classified as the otherside AOI. This classification was based exclusively on the stance measured before reading and did not depend on participants’ post-reading argument generation performance.

To quantify visual myside bias, difference scores were calculated separately for PTS and RFC:

PTS _difference_ = PTS _myside_ − PTS _otherside_

RFC _difference_ = RFC _myside_ − RFC _otherside_

A positive difference score indicated greater visual attention allocation or more frequent returns to the myside AOI than to the otherside AOI. A score of zero indicated no difference between the two AOIs, whereas a negative score indicated a greater value for the otherside AOI. These difference scores were used as indicators of visual myside bias in the subsequent correlation and regression analyses.

### 2.8. Data Analysis

Data were analyzed using IBM SPSS Statistics, version 26.0. Before the main analyses, the dataset was screened for missing values, outliers, and distributional characteristics. Descriptive statistics, including means, standard deviations, and ranges, were calculated for the study variables. Statistical significance was evaluated using a two-tailed α level of 0.05. Given the multiple statistical tests conducted across the research questions, the correlation and regression analyses were treated as exploratory. The reported *p* values were not adjusted for multiple comparisons and were interpreted alongside effect sizes, confidence intervals, and the consistency of the observed associations across measures. Findings with *p* values close to the conventional significance threshold were interpreted cautiously and should be confirmed in future studies.

To examine visual myside bias during reading, paired-samples t tests were conducted to compare percent time spent (PTS) and revisited fixation count (RFC) between the myside and otherside AOIs. Paired-samples effect sizes were expressed as Cohen’s d, calculated by dividing the mean paired difference by the standard deviation of the paired differences. To examine whether the observed differences were consistent across participants with different initial positions, the paired comparisons were repeated separately for participants who initially agreed and disagreed with the statement about the safety of genetically modified foods. Because the supporting and opposing panels occupied fixed left and right positions, these subgroup analyses were used to examine whether the myside–otherside differences were present in both stance groups.

Pearson’s correlation analyses were conducted to examine the relationships of prior knowledge, scientific epistemic beliefs, and positive and negative epistemic emotions with the two visual myside bias indicators and post-reading myside bias. The visual indicators were calculated as the difference between the myside and otherside AOIs for PTS and RFC, respectively. Pearson’s correlations were also used to examine the direct relationships between the two visual difference scores and the post-reading myside bias index. Correlation coefficients were reported with their corresponding *p* values and 95% confidence intervals.

Multiple linear regression analyses were conducted to examine the associations of prior knowledge, scientific epistemic beliefs, and negative epistemic emotions with visual and post-reading myside bias. Three separate models were specified, with PTS difference, RFC difference, and the post-reading myside bias index as the respective dependent variables. Prior knowledge, the overall scientific epistemic beliefs score, and negative epistemic emotions were entered simultaneously as predictors in each model. Unstandardized coefficients (B), standardized coefficients (β), standard errors, *p* values, and variance inflation factors (VIFs) were reported, together with the overall model statistics and adjusted R^2^.

To examine whether overall reading duration influenced the RFC difference score, total reading time was summarized using the mean, standard deviation, and range. Its association with the RFC difference score was examined using Pearson’s correlation. As a robustness check, total reading time was additionally entered as a covariate in the RFC regression model while retaining the original predictors. The coefficients and statistical significance of the original predictors were compared between the primary and adjusted models to assess whether the findings remained consistent after accounting for reading duration.

As a supplementary analysis, the original six-point pre-reading stance responses were retained to examine whether the magnitude of myside bias was associated with stance extremity. Stance extremity was calculated as the absolute distance between each participant’s initial stance score and the midpoint of the response scale (3.5). Spearman’s rank correlations were used to examine its relationships with PTS difference, RFC difference, and post-reading myside bias.

## 3. Results

### 3.1. Descriptive Statistics

Descriptive statistics for the study variables are presented in Table 1. The final sample comprised 57 participants. Based on their pre-reading stance toward the statement “Genetically modified foods are safe,” 22 participants (38.6%) initially agreed with the statement, whereas 35 participants (61.4%) initially disagreed. The distribution of responses on the original six-point stance scale was as follows: 1 (*n* = 3), 2 (*n* = 15), 3 (*n* = 17), 4 (*n* = 18), 5 (*n* = 4), and 6 (*n* = 0).

Participants generated an average of 5.56 valid arguments (SD = 1.24), with scores ranging from 3 to 9. The mean post-reading myside bias index was 1.07 (SD = 0.96, range = 0–4). Eighteen participants had a bias index of 0, indicating that they generated equal numbers of myside and otherside arguments. The remaining participants had positive bias indices: 22 scored 1, 13 scored 2, three scored 3, and one scored 4. No participant generated more otherside than myside arguments.

For the eye-tracking measures, mean PTS was 35.69% (SD = 2.67%) for the myside AOI and 29.28% (SD = 2.28%) for the otherside AOI. The mean PTS difference was 6.41 percentage points (SD = 3.60). Mean RFC was 46.65 (SD = 3.36) for the myside AOI and 37.37 (SD = 3.18) for the otherside AOI, with a mean difference of 9.28 (SD = 4.63). Total reading time was 108.55 s (SD = 47.05 s). The eye-tracking measures showed relatively narrow between-participant ranges. This restricted dispersion may partly reflect the highly standardized reading stimulus and the use of large, fixed AOIs with identical spatial dimensions across participants. In addition, RFC was derived at the AOI level after fixation events were processed using the same Tobii I-VT settings for all recordings.

### 3.2. Visual Attention to Myside and Otherside Information

To address Research Question 1, paired-samples t tests were conducted to compare participants’ PTS and RFC between the myside and otherside AOIs. The results are presented in Table 2. Participants spent a significantly greater proportion of viewing time on myside information (M = 35.69%, SD = 2.67%) than on otherside information (M = 29.28%, SD = 2.28%), t(56) = 13.42, *p* < 0.001, Cohen’s d = 1.78. Similarly, RFC was significantly higher for the myside AOI (M = 46.65, SD = 3.36) than for the otherside AOI (M = 37.37, SD = 3.18), t(56) = 15.13, *p* < 0.001, Cohen’s d = 2.00. These results indicate that participants allocated a greater proportion of viewing time to information consistent with their initial stance and returned to that information more frequently during reading.

To examine whether the observed myside–otherside differences were present in both initial stance groups, separate paired-samples t tests were conducted for participants who initially agreed and disagreed with the statement that genetically modified foods are safe. For participants who initially agreed (*n* = 22), PTS was significantly higher for the myside AOI (M = 35.14%, SD = 2.89%) than for the otherside AOI (M = 29.68%, SD = 2.60%), t(21) = 5.94, *p* < 0.001, Cohen’s d = 1.27. RFC was also significantly higher for the myside AOI (M = 46.14, SD = 4.05) than for the otherside AOI (M = 38.05, SD = 3.26), t(21) = 7.14, *p* < 0.001, Cohen’s d = 1.52.

A similar pattern was observed among participants who initially disagreed (*n* = 35). PTS was significantly higher for the myside AOI (M = 36.03%, SD = 2.51%) than for the otherside AOI (M = 29.04%, SD = 2.06%), t(34) = 13.84, *p* < 0.001, Cohen’s d = 2.34. RFC was also significantly higher for the myside AOI (M = 46.97, SD = 2.85) than for the otherside AOI (M = 36.94, SD = 3.11), t(34) = 14.66, *p* < 0.001, Cohen’s d = 2.48. The subgroup results are presented in Table 3. The individual distributions of PTS and RFC for the myside and otherside AOIs are shown in Figure 3.

The myside AOI received significantly higher PTS and RFC values in both initial stance groups, although the myside information appeared on opposite sides of the screen for the two groups. This pattern is inconsistent with an explanation based solely on a uniform left- or right-side preference.

To further examine whether the magnitude of visual myside bias differed between the initial stance groups, Welch’s independent-samples t tests were conducted using the PTS and RFC difference scores. The PTS difference score did not differ significantly between participants who initially agreed (M = 5.47, SD = 4.32) and those who initially disagreed (M = 7.00, SD = 2.99), t(33.67) = −1.46, *p* = 0.155, 95% CI [−3.67, 0.61]. Similarly, the RFC difference score did not differ significantly between the agreeing group (M = 8.09, SD = 5.32) and the disagreeing group (M = 10.03, SD = 4.05), t(36.12) = −1.46, *p* = 0.152, 95% CI [−4.62, 0.75]. Thus, although significant myside–otherside differences were observed within both initial stance groups, the analyses did not detect a significant between-group difference in the magnitude of visual myside bias.

As a supplementary analysis, Spearman’s rank correlations were conducted to examine whether stance extremity was associated with the magnitude of visual and post-reading myside bias. Stance extremity was not significantly correlated with PTS difference (ρ = 0.078, *p* = 0.563), RFC difference (ρ = 0.057, *p* = 0.675), or post-reading myside bias (ρ = −0.035, *p* = 0.794). These analyses did not detect a significant monotonic association between the extremity of participants’ initial stance and any of the three myside bias indicators.

To further describe the distribution of myside bias across levels of initial stance strength, participants were grouped according to their distance from the midpoint of the six-point stance scale. Participants selecting 3 or 4 were classified as having a weak, near-midpoint stance (*n* = 35), those selecting 2 or 5 as having a moderate stance (*n* = 19), and those selecting 1 or 6 as having a strong stance (*n* = 3). As shown in Appendix H, the mean PTS difference was 6.18 (SD = 3.95) in the weak-stance group, 6.76 (SD = 3.06) in the moderate-stance group, and 6.77 (SD = 3.48) in the strong-stance group. The corresponding mean RFC differences were 9.34 (SD = 5.05), 9.11 (SD = 4.08), and 9.67 (SD = 4.16), respectively. Mean post-reading myside bias was likewise similar across the three groups (1.11, 1.00, and 1.00, respectively). These descriptive results indicate that the observed myside pattern was also present among participants whose initial stance responses were closest to the scale midpoint and was not confined to participants holding stronger initial positions.

### 3.3. Associations Between Visual and Post-Reading Myside Bias

To address Research Question 2, Pearson’s correlation analyses were conducted to examine the associations between visual myside bias during reading and post-reading myside bias in argument generation. Visual myside bias was represented by the PTS and RFC difference scores, whereas post-reading myside bias was calculated as the difference between the numbers of myside and otherside arguments generated after reading. The results are presented in Table 4. The PTS difference score was positively correlated with post-reading myside bias, r(55) = 0.778, *p* < 0.001, 95% CI [0.649, 0.863]. Similarly, the RFC difference score was positively correlated with post-reading myside bias, r(55) = 0.738, *p* < 0.001, 95% CI [0.591, 0.837].

These results indicate that participants who allocated a greater proportion of viewing time to myside information than to otherside information also tended to generate more arguments consistent with their initial stance after reading. A similar association was observed for the frequency of returns to the myside AOI relative to the otherside AOI.

### 3.4. Predictors of Visual Myside Bias

To address Research Question 3, Pearson’s correlation analyses were conducted to examine the associations of prior knowledge, scientific epistemic beliefs, and positive and negative epistemic emotions with visual myside bias during SSI reading. Visual myside bias was operationalized using the PTS and RFC difference scores, calculated by subtracting the otherside AOI value from the corresponding myside AOI value. The results are presented in Table 5.

To examine the associations of the cognitive and affective variables with visual myside bias while accounting for their shared variance, two multiple linear regression models were estimated. Prior knowledge, scientific epistemic beliefs, and negative epistemic emotions were entered simultaneously as predictors, with PTS difference and RFC difference serving as the dependent variables in separate models. Positive epistemic emotions were not included in these models, consistent with the original regression specification.

The regression model for PTS difference was statistically significant, F(3, 53) = 15.653, *p* < 0.001, with an adjusted R^2^ of 0.440. As shown in Table 6, prior knowledge was a significant negative predictor of PTS difference (B = −1.185, β = −0.450, *p* < 0.001), as were scientific epistemic beliefs (B = −6.631, β = −0.271, *p* = 0.026). Negative epistemic emotions were not a significant predictor after accounting for the other variables (B = 0.167, β = 0.115, *p* = 0.403). Prior knowledge had a larger absolute standardized regression coefficient than scientific epistemic beliefs in this model.

The regression model for RFC difference was also statistically significant, F(3, 53) = 14.045, *p* < 0.001, with an adjusted R^2^ of 0.411. As presented in Table 7, prior knowledge (B = −1.470, β = −0.429, *p* = 0.001) and scientific epistemic beliefs (B = −8.127, β = −0.255, *p* = 0.039) were significant negative predictors of RFC difference. Negative epistemic emotions were not significantly associated with RFC difference after controlling for the other predictors (B = 0.238, β = 0.127, *p* = 0.370). As in the PTS model, prior knowledge had a larger absolute standardized regression coefficient than scientific epistemic beliefs.

To examine whether visual myside bias was associated with overall reading duration, Pearson’s correlation analyses were conducted using total reading time (TRT). TRT was not significantly correlated with RFC difference, r(55) = −0.019, *p* = 0.886, 95% CI [−0.279, 0.242], or PTS difference, r(55) = −0.071, *p* = 0.602, 95% CI [−0.325, 0.194].

A supplementary multiple linear regression analysis was conducted to assess whether the associations observed in the RFC difference model remained consistent after accounting for TRT. The extended model included prior knowledge, scientific epistemic beliefs, negative epistemic emotions, and TRT as simultaneous predictors. The overall model was statistically significant, F(4, 52) = 10.338, *p* < 0.001, R^2^ = 0.443, adjusted R^2^ = 0.400. TRT was not a significant predictor of RFC difference (B = 0.00084, β = 0.008, *p* = 0.935), and its inclusion resulted in a negligible increase in explained variance (ΔR^2^ < 0.001). The standardized regression coefficients for prior knowledge (β = −0.430, *p* = 0.002) and scientific epistemic beliefs (β = −0.256, *p* = 0.041) remained similar to those in the primary model, whereas negative epistemic emotions remained nonsignificant (β = 0.128, *p* = 0.373). The full results of the extended model are presented in Appendix E.

### 3.5. Predictors of Post-Reading Myside Bias

To address Research Question 4, Pearson’s correlation analyses were conducted to examine the associations of prior knowledge, scientific epistemic beliefs, and positive and negative epistemic emotions with post-reading myside bias in argument generation. Post-reading myside bias was calculated by subtracting the number of otherside arguments from the number of myside arguments generated after reading. The results are presented in Table 8.

Positive epistemic emotions were not significantly correlated with post-reading myside bias, r(55) = 0.123, *p* = 0.361. In contrast, negative epistemic emotions were positively correlated with post-reading myside bias, r(55) = 0.591, *p* < 0.001. Scientific epistemic beliefs, r(55) = −0.594, *p* < 0.001, and prior knowledge, r(55) = −0.716, *p* < 0.001, were both negatively correlated with post-reading myside bias. Thus, higher prior knowledge and scientific epistemic beliefs scores were associated with smaller differences between the numbers of myside and otherside arguments, whereas higher negative epistemic emotions scores were associated with larger differences.

A multiple linear regression analysis was subsequently conducted to examine the associations of prior knowledge, scientific epistemic beliefs, and negative epistemic emotions with post-reading myside bias while accounting for their shared variance. The three variables were entered simultaneously as predictors, with post-reading myside bias as the dependent variable. Positive epistemic emotions were not included in the model, consistent with the regression specification used for the visual myside bias analyses.

The regression model was statistically significant, F(3, 53) = 32.441, *p* < 0.001, R^2^ = 0.647, adjusted R^2^ = 0.627. As shown in Table 9, prior knowledge was a significant negative predictor of post-reading myside bias (B = −0.378, β = −0.538, *p* < 0.001), as were scientific epistemic beliefs (B = −2.340, β = −0.358, *p* < 0.001). Negative epistemic emotions were not a significant predictor after accounting for the other variables (B = 0.032, β = 0.083, *p* = 0.459). The absolute standardized regression coefficient was larger for prior knowledge than for scientific epistemic beliefs. Additional diagnostics indicated no significant departure from residual normality or homoscedasticity, although the model specification test and fitted-value range suggested limitations of the linear model. Sensitivity analyses using HC3 robust standard errors and an influence-point check retained the main pattern of associations.

### 3.6. Sensitivity Analysis of the Prior Knowledge Measure

Because Items 4 and 10 showed negative corrected item–total correlations, a sensitivity analysis was conducted using a reduced prior knowledge score with these two items removed. The KR-20 coefficient increased from 0.603 for the original 10-item score to 0.662 for the reduced eight-item score (Appendix G).

Accordingly, the correlations between prior knowledge and PTS difference, RFC difference, and post-reading myside bias were unchanged, remaining r = −0.615, r = −0.571, and r = −0.716, respectively (all *p* < 0.001). The corresponding multiple regression results were also unchanged with respect to the regression slopes, standardized coefficients, significance levels, and model fit. Because Items 4 and 10 showed negative corrected item–total correlations, a reduced prior knowledge score excluding these two items was examined. The KR-20 coefficient increased from 0.603 for the original 10-item score to 0.662 for the reduced eight-item score. However, the removal of Items 4 and 10 reduced each participant’s score by approximately one point while leaving the standard deviation and rank ordering unchanged. Consequently, the correlations with PTS difference, RFC difference, and post-reading myside bias remained numerically identical. This invariance reflects the near-constant contribution of the two excluded items rather than providing an independent test of the robustness of the associations.

## 4. Discussion

This study examined visual myside bias during the reading of conflicting SSI texts about genetically modified foods and its association with post-reading myside bias in argument generation. It also investigated the relationships of prior knowledge, scientific epistemic beliefs, and epistemic emotions with these two forms of bias. The results showed that participants allocated a greater proportion of viewing time to myside information and returned to it more frequently than to otherside information. Visual myside bias was positively associated with post-reading myside bias: participants who showed larger differences in visual attention between myside and otherside information also tended to generate relatively more arguments consistent with their initial positions. In addition, prior knowledge and scientific epistemic beliefs were negatively associated with both visual and post-reading myside bias in the primary regression models. Although negative epistemic emotions were positively correlated with the bias indicators, their regression coefficients were not statistically significant after accounting for the cognitive variables.

### 4.1. Visual Myside Bias as Stance-Consistent Processing

For Research Question 1, this study found that college students showed significantly higher percent time spent (PTS) and revisited fixation count (RFC) in the myside area than in the otherside area when reading the SSI text on genetically modified foods. This indicates that students allocated more visual attention to information consistent with their initial positions and returned to this information more frequently during reading. The result suggests that myside bias can be observed not only in post-reading argument generation but also in visual attention allocation during reading. This finding is generally consistent with previous studies on controversial issue reading and selective processing. Myside bias is often reflected in individuals’ tendency to attend to, accept, or generate information that supports their own positions, while paying less attention to or evaluating less favorably information that challenges their positions [2,5,7,10]. In the present study, students’ visual attention was not evenly distributed between myside and otherside information. Rather, it was more concentrated on text areas consistent with their own positions. Higher PTS indicates that students spent a greater proportion of viewing time in the myside area, while higher RFC indicates that they returned to this area more frequently.

The subgroup analyses provided further evidence relevant to the interpretation of these findings. Participants who initially agreed and those who initially disagreed with the safety of GM foods both showed significantly higher PTS and RFC in the myside area than in the otherside area. Because supporting information was consistently presented on the left and opposing information on the right, the myside area was located on opposite sides of the screen for the two groups. The presence of myside–otherside differences in both groups suggests that the overall pattern cannot be readily explained by a uniform preference for one side of the screen. Nevertheless, the panel positions were not counterbalanced, and possible effects of spatial layout and differences in textual content cannot be completely excluded.

However, higher PTS and RFC in the myside area should not be interpreted simply as higher-quality reasoning or more sufficient critical reading. Previous eye-tracking studies suggest that longer viewing time and more revisits can reflect greater attention investment or repeated examination of information, but these indicators do not directly reveal the purpose or quality of processing [54,55,56,57,58,59,60]. In SSI reading involving conflicting information, the same eye-tracking pattern may have different meanings. Greater attention to myside information may reflect position-consistent processing, but it may also be associated with familiarity, comprehension difficulty, or other reading processes. Research on the text-belief consistency effect and motivated processing offers one possible explanation for such attention differences. Maier and Richter’s two-step validation model suggests that readers evaluate information in relation to their existing knowledge and beliefs, and that prior beliefs may influence the validation of conflicting information [21,61,62]. Thus, the greater visual attention to myside information observed in this study does not necessarily indicate more open or balanced evidence evaluation, nor can the eye-tracking measures alone establish that participants deliberately sought to strengthen their initial positions.

Regarding Research Question 2, both the PTS difference score and the RFC difference score were positively associated with post-reading myside bias. Participants who allocated relatively more visual attention to myside information during reading also tended to generate relatively more arguments consistent with their initial positions afterward. This finding suggests that stance-consistent attention during reading and stance-consistent argument generation after reading are related aspects of students’ responses to conflicting SSI information. Nevertheless, the observed associations do not establish that greater attention to myside information caused participants to generate more myside arguments. The two measures capture different aspects of information processing, and their relationship may also reflect individual differences that were not directly examined in this study.

An important theoretical implication of this study is that it highlights the need to distinguish between the amount of visual attention and the quality of epistemic processing when interpreting eye-tracking indicators. Longer viewing time and more revisits show that students allocated more visual attention to certain types of information. They do not establish that the processing was necessarily critical, balanced, or evidence-oriented. For SSI controversial text reading, the key issue is therefore not simply whether students engaged in more processing, but which information they attended to and how they considered evidence from different positions [39]. The positive associations between visual myside bias and post-reading myside bias further suggest that attention allocation during reading is relevant to understanding subsequent argument generation. However, determining whether such attention facilitates critical evaluation or reinforces an existing position requires evidence beyond the eye-tracking measures used in the present study.

### 4.2. Cognitive and Affective Predictors of Online and Offline Myside Bias

For Research Questions 3 and 4, this study examined the associations of scientific epistemic beliefs, prior knowledge, and epistemic emotions with visual myside bias during reading and post-reading myside bias. The results showed a generally consistent pattern across the two forms of bias. Scientific epistemic beliefs and prior knowledge were negatively associated with visual myside bias and post-reading myside bias in the primary regression models. Although negative epistemic emotions were positively correlated with both forms of bias, their regression coefficients were not statistically significant after accounting for scientific epistemic beliefs and prior knowledge. Positive epistemic emotions were not significantly correlated with either form of bias. These findings suggest that individual differences in cognitive characteristics are associated with stance-consistent attention allocation during reading as well as the balance of arguments generated afterward.

The relationships observed across the two forms of bias extend the examination of myside bias beyond post-reading outcomes. Visual myside bias reflects how students allocate attention to information consistent or inconsistent with their initial positions, whereas post-reading myside bias reflects the relative numbers of arguments they generate for the two positions. The negative associations of scientific epistemic beliefs and prior knowledge with both measures suggest that these cognitive characteristics may be relevant to students’ engagement with conflicting SSI information at different stages of the reading task. However, the present findings do not establish that these characteristics directly influence visual attention or subsequent argument generation, or that the two forms of bias arise from a single underlying mechanism.

Previous research on myside bias suggests that it is relevant to rational thinking and is not adequately explained by general intelligence or traditional cognitive ability alone [10,52,63]. Scientific epistemic beliefs may also be relevant to how students approach competing claims and consider evidence that challenges their existing positions [46,50]. In the present study, higher scientific epistemic beliefs scores were associated with smaller visual and post-reading myside bias scores. One possible explanation is that students who place greater emphasis on the development and evidential justification of scientific knowledge may be more willing to consider information from different positions. Nevertheless, the overall scientific epistemic beliefs score combined four dimensions, and the present analyses do not establish whether each dimension showed the same relationship with myside bias. The interpretation of the composite score should also take account of its limited internal consistency.

Prior knowledge showed a larger absolute standardized regression coefficient than scientific epistemic beliefs in each of the three primary models. One possible explanation is that SSI involve scientific knowledge, social factors, and value judgments. Prior knowledge may support belief-consistent processing, but it may also provide cognitive resources for understanding conflicting evidence [9,21,64,65]. Students with more topic knowledge may be better able to understand the concepts, evidence, and risk explanations presented by both sides and may therefore rely less on information consistent with their initial positions when reading and generating arguments. In contrast, students with less prior knowledge may experience greater difficulty understanding unfamiliar or opposing information. These interpretations are consistent with the observed negative associations, but the present study cannot determine whether greater prior knowledge itself reduces myside bias.

However, the association involving prior knowledge should be interpreted cautiously because the 10-item prior knowledge measure showed limited internal consistency (KR-20 = 0.603), several items had extreme difficulty levels, and the observed total score range was restricted. In this sample, meaningful between-participant discrimination was effectively provided by only five items (Items 5–9), which further limits the precision with which prior knowledge was measured. Although the sensitivity analysis excluding the two negatively discriminating items yielded the same pattern of associations, replication with a more discriminating and psychometrically stronger measure of topic-specific prior knowledge is needed.

Negative epistemic emotions were positively correlated with visual myside bias and post-reading myside bias. Students who retrospectively reported more negative emotions during reading tended to show larger differences in attention to myside and otherside information and to generate relatively more myside arguments afterward. Such associations may be considered in relation to emotional responses to conflicting information [55,66]. However, negative epistemic emotions were not statistically significant predictors in the regression models after prior knowledge and scientific epistemic beliefs were taken into account. Their associations with myside bias may therefore overlap with those of the cognitive variables examined in this study. The findings do not establish an interaction between emotions and cognitive characteristics, nor do they demonstrate that negative emotions independently produce stance-consistent processing. Furthermore, because epistemic emotions were reported retrospectively after reading, their temporal relationship with visual attention cannot be determined from the present data.

The supplementary analysis further showed that including total reading time in the RFC difference score regression model did not materially change the estimated associations of prior knowledge and scientific epistemic beliefs with visual myside bias. This finding suggests that the associations observed in the primary RFC model were not explained solely by differences in overall reading duration. Nevertheless, other characteristics of the reading process may also be relevant to revisit behavior.

Taken together, these findings suggest that supporting students’ engagement with conflicting SSI information may require attention to both their topic knowledge and their understanding of scientific evidence. Encouraging students to consider arguments from opposing positions, examine the justification for competing claims, and recognize uncertainty in scientific knowledge may provide useful directions for SSI reading instruction. However, the present study did not test an instructional intervention, and whether such approaches reduce visual or post-reading myside bias requires further investigation.

## 5. Limitations and Future Research

The present study has several limitations. First, the sample size was modest and consisted only of college students, which limits the generalizability of the findings and the precision of the estimated associations. Future studies should replicate the findings with larger samples and participants from different age groups. Second, the study focused on a single SSI topic; namely, genetically modified foods. Because myside bias and epistemic beliefs can be topic-specific, future research should examine whether similar patterns occur in other SSI contexts, such as vaccination, climate change, and nuclear energy [41,67].

Third, the distribution of pre-reading stance responses was concentrated near the midpoint of the six-point scale. Thirty-five of the 57 participants (61.4%) selected response options 3 or 4, meaning that their classification as initially disagreeing or agreeing depended on a relatively narrow difference around the scale midpoint. Although the supplementary analyses did not indicate that the observed myside pattern was confined to participants with stronger initial positions, this compressed stance distribution limits the extent to which the findings can be generalized to individuals holding clearly polarized positions. Future studies should include samples with a wider distribution of initial stance strength and may also consider analyzing stance as a continuous variable. The prior knowledge measure also showed limited internal consistency (KR-20 = 0.603) and a restricted score range. Items 1–3 showed no between-participant variance, and Items 4 and 10 contributed little discriminating information, such that meaningful score variation was effectively determined by five items (Items 5–9). Because removing Items 4 and 10 primarily shifted the total score by a near-constant amount, the corresponding sensitivity analysis could not provide an independent test of the robustness of the observed associations. Future studies should use a more discriminating measure of topic-specific knowledge with stronger psychometric properties.

Fourth, the supporting and opposing text panels were analyzed as broad areas of interest (AOIs). This approach was suitable for examining panel-level attention allocation but did not permit conclusions about attention to individual claims or specific pieces of evidence. In addition, the positions of the supporting and opposing panels were not counterbalanced. Although both the initially agreeing and initially disagreeing groups showed greater visual attention to myside information, potential effects of spatial layout and differences in textual content cannot be completely excluded. Future studies should counterbalance panel positions and consider more fine-grained AOIs to examine how readers attend to specific claims and evidence.

Fifth, epistemic emotions were assessed retrospectively after reading. Participants’ reports may therefore have been influenced by their recall or subsequent evaluation of the reading experience. Consequently, the temporal relationship between their reported emotions and visual attention during reading could not be established. Future studies could use measures collected at multiple points during the reading task to examine changes in epistemic emotions and their associations with attention allocation.

In addition, the exploratory correlation and regression analyses involved multiple statistical tests without adjustment for multiple comparisons, which may increase the risk of false-positive findings. Future studies with prespecified hypotheses and larger samples are needed to confirm the observed associations.

Finally, the correlational design does not allow causal conclusions about whether visual myside bias leads to post-reading myside bias. The two measures should be interpreted as associated manifestations of stance-consistent processing rather than as a confirmed causal sequence. Future experimental studies could manipulate reading conditions or attention guidance strategies to examine whether changes in attention allocation are accompanied by changes in subsequent argument generation.

## 6. Conclusions and Implications

This study examined visual myside bias during the reading of conflicting SSI texts about genetically modified foods and its association with post-reading myside bias in argument generation. Participants allocated a greater proportion of viewing time to myside information and returned to it more frequently than to otherside information. Both visual attention difference scores were positively associated with post-reading myside bias, indicating that participants who showed greater stance-consistent attention during reading also tended to generate relatively more arguments supporting their initial positions afterward. In addition, prior knowledge and scientific epistemic beliefs were negatively associated with both visual and post-reading myside bias in the primary regression models. Although negative epistemic emotions were positively correlated with the bias indicators, their regression coefficients were not statistically significant after accounting for the cognitive variables.

These findings have implications for SSI reading instruction and the interpretation of eye-tracking measures. Longer viewing time and more frequent revisits should not be taken as direct evidence of higher-quality critical reasoning, because these measures indicate visual attention rather than the quality of evidence evaluation. In SSI reading activities, educators may therefore consider not only whether students engage with conflicting texts, but also whether they attend to information that challenges their initial positions. Providing relevant background knowledge and encouraging students to compare competing claims, examine the evidence supporting each position, and consider opposing arguments may offer useful directions for instruction. However, the present study did not evaluate an instructional intervention, and further research is needed to determine whether these approaches can reduce visual or post-reading myside bias.

## Figures and Tables

**Figure 1:**
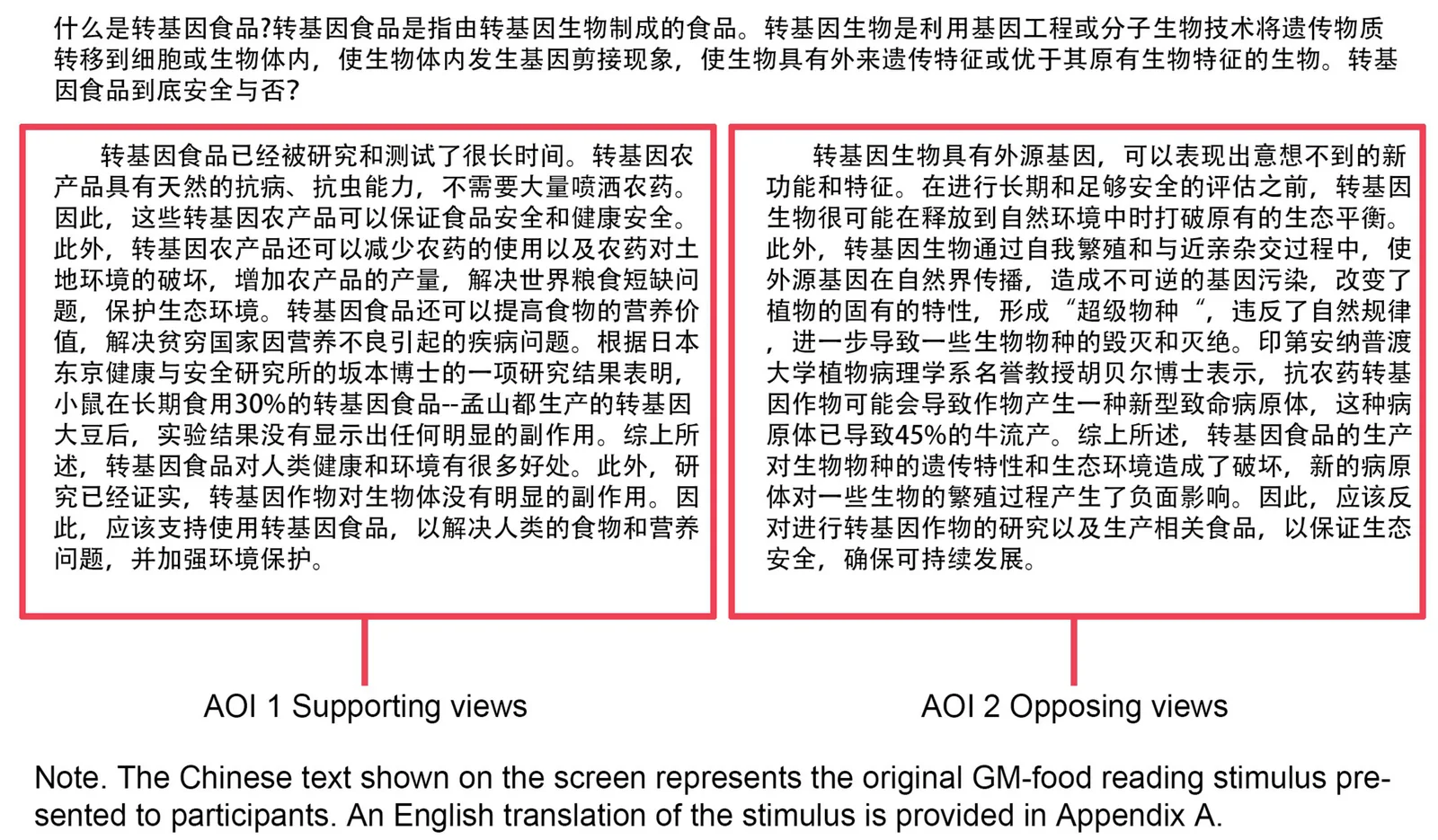
Reading materials and the division of AOIs.

**Figure 2:**
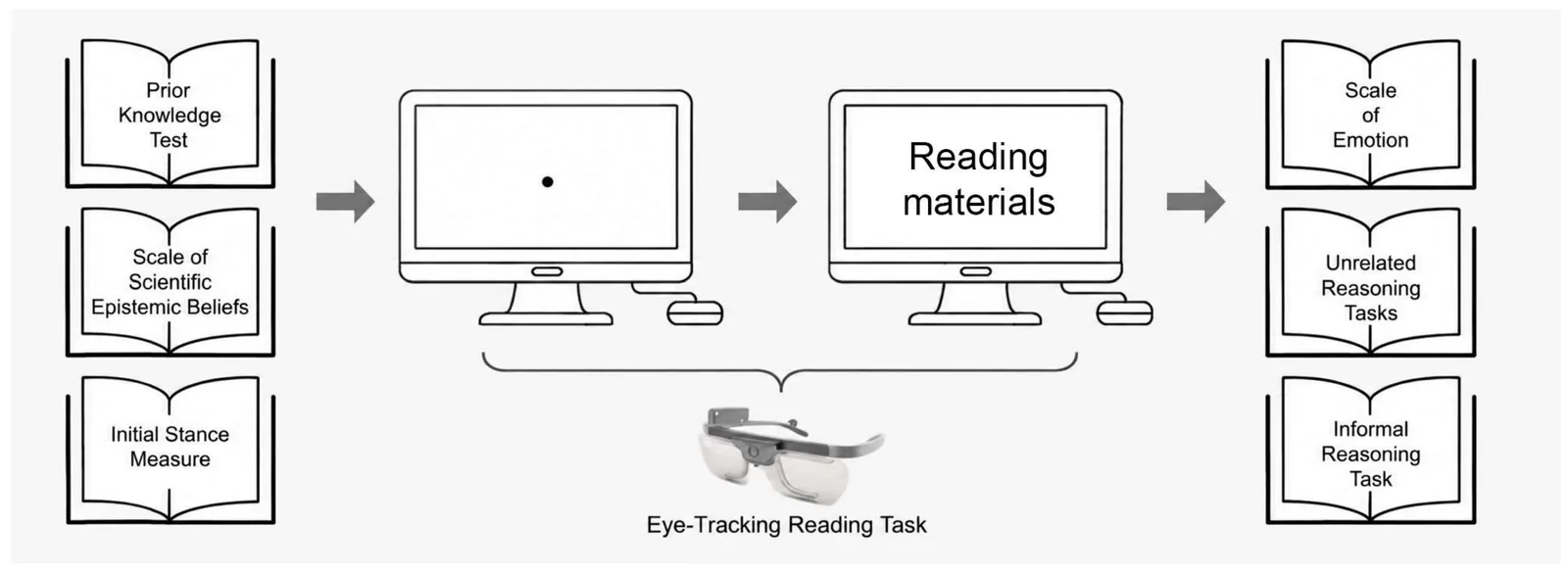
Experiment procedure.

**Figure 3:**
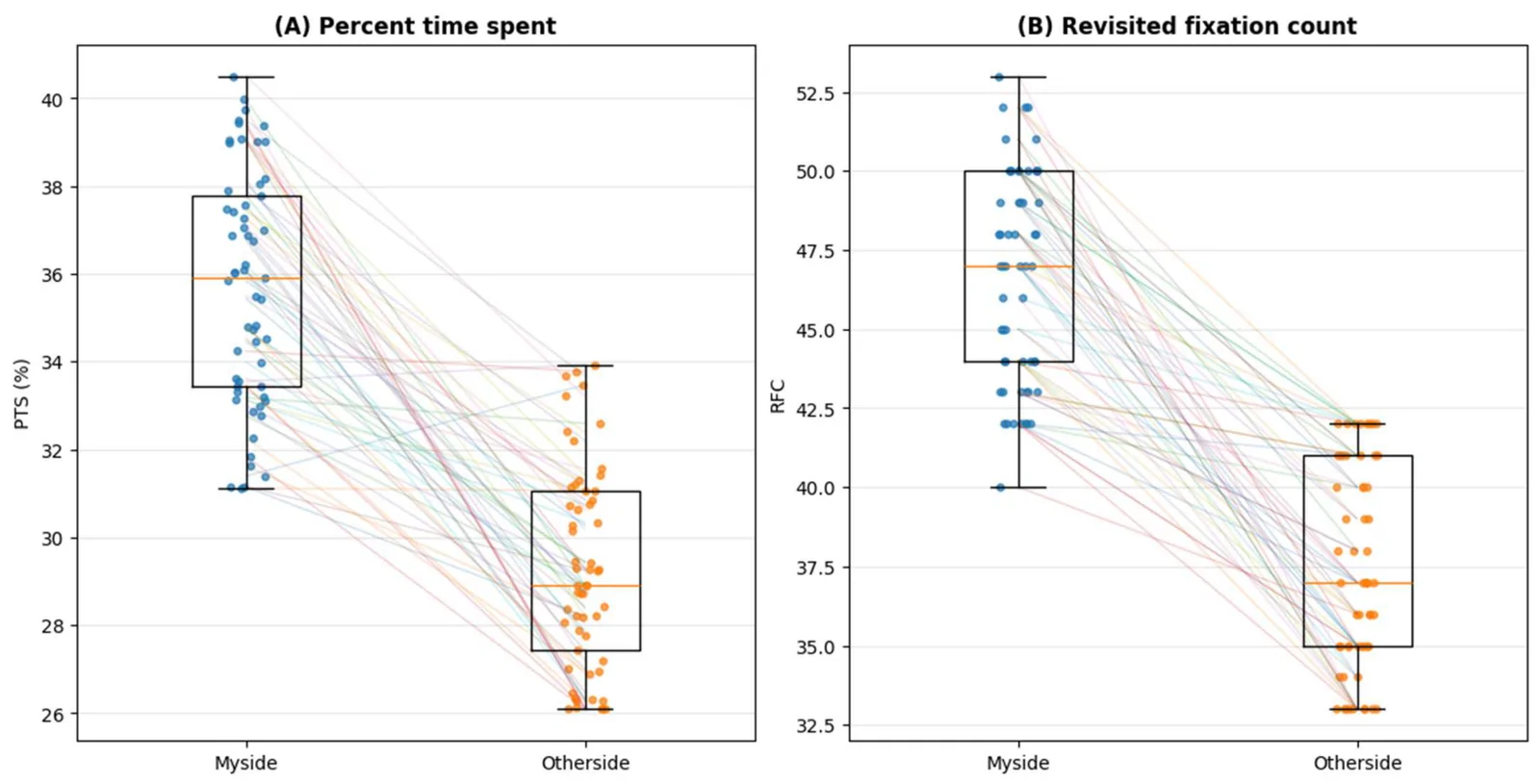
Individual distributions of percent time spent (**A**) and revisited fixation count (**B**) for myside and otherside areas of interest.

**Table 1:** Results of Descriptive statistics.

Variables	M	SD	Min	Max
Valid argument count	5.56	1.24	3	9
Post-reading myside bias	1.07	0.96	0	4
Positive epistemic emotions	20.84	3.11	16	25
Negative epistemic emotions	17.93	2.49	13	23
Scientific epistemic beliefs	3.98	0.15	3.6	4.28
Prior knowledge	6.67	1.37	4	9
Myside PTS (%)	35.69	2.67	31.11	40.51
Otherside PTS (%)	29.28	2.28	26.09	33.93
PTS difference (percentage points)	6.41	3.6	−2.08	13.66
Myside RFC	46.65	3.36	40	53
Otherside RFC	37.37	3.18	33	42
RFC difference	9.28	4.63	1	20
Total reading time (s)	108.55	47.05	31.85	250.05

**Table 2:** Results of eye-tracking metrics for myside and otherside areas.

Measure	Mean Difference (SD)	95% CI	t	*p*	Cohen’s d
PTS (%)	6.41 (3.60)	[5.45, 7.36]	13.42	<0.001	1.78
RFC	9.28 (4.63)	[8.05, 10.51]	15.13	<0.001	2.00

**Table 3:** Paired-Samples Comparisons of Visual Attention by Initial Stance.

Initial Stance	Measure	Myside M (SD)	Otherside M (SD)	t	*p*	Cohen’s d
Agree (*n* = 22)	PTS (%)	35.14 (2.89)	29.68 (2.60)	5.94	<0.001	1.27
Agree (*n* = 22)	RFC	46.14 (4.05)	38.05 (3.26)	7.14	<0.001	1.52
Disagree (*n* = 35)	PTS (%)	36.03 (2.51)	29.04 (2.06)	13.84	<0.001	2.34
Disagree (*n* = 35)	RFC	46.97 (2.85)	36.94 (3.11)	14.66	<0.001	2.48

**Table 4:** Correlations Between Visual and Post-Reading Myside Bias (N = 57).

Visual Myside Bias Indicator	Pearson’s r (df = 55)	95% CI	*p*
PTS difference	0.778	[0.649, 0.863]	<0.001
RFC difference	0.738	[0.591, 0.837]	<0.001

**Table 5:** Correlations of Cognitive and Affective Variables With Visual Myside Bias.

Predictor	PTS Difference r (df = 55)	95% CI	*p*	RFC Difference r (df = 55)	95% CI	*p*
Positive epistemic emotions	0.059	[−0.205, 0.314]	0.664	0.051	[−0.212, 0.308]	0.704
Negative epistemic emotions	0.524	[0.306, 0.691]	<0.001	0.568	[0.361, 0.722]	<0.001
Scientific epistemic beliefs	−0.492	[−0.667, −0.265]	<0.001	−0.449	[−0.635, −0.213]	<0.001
Prior knowledge	−0.615	[−0.754, −0.422]	<0.001	−0.571	[−0.724, −0.365]	<0.001

**Table 6:** Multiple Regression Analysis Predicting PTS Difference.

Predictor	B	SE	β	*p*	VIF
Constant	37.705	13.71	—	0.008	—
Prior knowledge	−1.185	0.329	−0.450	<0.001	1.552
Scientific epistemic beliefs	−6.631	2.886	−0.271	0.026	1.387
Negative epistemic emotions	0.167	0.198	0.115	0.403	1.87

**Table 7:** Multiple Regression Analysis Predicting RFC Difference.

Predictor	B	SE	β	*p*	VIF
Constant	47.304	18.246	—	0.012	—
Prior knowledge	−1.470	0.437	−0.429	0.001	1.552
Scientific epistemic beliefs	−8.127	3.841	−0.255	0.039	1.387
Negative epistemic emotions	0.238	0.263	0.127	0.37	1.87

**Table 8:** Correlations of Cognitive and Affective Variables with Post-Reading Myside Bias.

Predictor	Pearson’s r (df = 55)	95% CI	*p*
Positive epistemic emotions	0.123	[−0.142, 0.372]	0.361
Negative epistemic emotions	0.591	[0.391, 0.738]	<0.001
Scientific epistemic beliefs	−0.594	[−0.740, −0.395]	<0.001
Prior knowledge	−0.716	[−0.823, −0.560]	<0.001

**Table 9:** Multiple Regression Analysis Predicting Post-Reading Myside Bias.

Predictor	B	SE	β	95% CI for B	*p*	VIF
Constant	12.327	2.981	—	[6.349, 18.306]	<0.001	—
Prior knowledge	−0.378	0.071	−0.538	[−0.522, −0.235]	<0.001	1.552
Scientific epistemic beliefs	−2.340	0.628	−0.358	[−3.598, −1.081]	<0.001	1.387
Negative epistemic emotions	0.032	0.043	0.083	[−0.054, 0.118]	0.459	1.87

## Data Availability

The data presented in this study are available from the corresponding author upon reasonable request. The data are not publicly available because they contain participant-level research data.

## Appendix A. Contradictory Article (English Version)

What are genetically modified (GM) foods? GM foods are foods made from genetically modified organisms (GMOs). GMOs are organisms in which genetic material has been altered using genetic engineering or molecular biology techniques, resulting in gene splicing within cells or organisms to give the organism specific traits or improved characteristics over its original state. Are GM foods safe?

GM foods have been researched and tested for a long time. GM crops have natural resistance to diseases and pests, reducing the need for extensive pesticide spraying. Therefore, these GM crops can ensure food safety and health. Additionally, GM crops can reduce pesticide use and the damage pesticides cause to soil environments, increase crop yields, address world food shortages, and protect the ecological environment. GM foods can also enhance the nutritional value of food and address the problem of diseases caused by malnutrition in poor countries. According to a study by Dr. Sakamoto of the Tokyo Institute of Health and Safety, mice fed with 30% GM soybeans produced by Monsanto for a long period (52 and 104 weeks) showed no significant side effects. In conclusion, GM foods have many benefits for human health and the environment. Furthermore, studies have shown that GM crops have no significant adverse effects on organisms. Therefore, the use of GM foods should be supported to address human food and nutrition issues and strengthen environmental protection.

GM organisms contain foreign genes and can exhibit unexpected new functions and characteristics. Without long-term and sufficiently safe assessments, GM organisms are likely to disrupt the existing ecological balance when released into the natural environment. Additionally, GM organisms spread the foreign genes in the natural environment through self-reproduction and crossbreeding with close relatives, causing irreversible genetic pollution, altering the inherent characteristics of plants, forming “super species,” violating natural laws, and further leading to the destruction and extinction of some species. Dr. Huber, Professor Emeritus of Plant Pathology at Purdue University, has stated that GM crops resistant to pesticides may lead to the development of a new deadly pathogen in crops, which has caused 45% of cows to miscarry. In summary, the production of GM foods has caused destruction to the genetic characteristics of biological species and the ecological environment, and the new pathogen has had a negative impact on the reproductive process of some organisms. Therefore, research and production of GM crops and related foods should be opposed.

Note. The passages reproduced here are English translations of the experimental stimulus presented to participants in Chinese. The stimulus was adapted from Chinese-language popular science and debate materials for experimental purposes. Some factual claims in the passages reflect contested claims that circulated in public debate and should not be interpreted as claims endorsed by the authors.

## Appendix B. Prior Knowledge Test

Below are statements about genetically modified foods. Please choose the answer that is the most consistent with your own knowledge.

1. Genetically modifying foods occurs through …
  - natural processes.
  - artificial processes.
  - all of the above
  - none of the above
2. Processes used by scientists to modify the genetic makeup of plants and animals include which of the following?
  - Exact replica cloning
  - Hormone injection
  - Cross-pollination
  - All of the above
3. When using gene cloning methods a genetically modified organism is …
  - an exact replica of the donor organism.
  - a bit different than the donor organism.
  - in no way similar to the donor organism.
  - Gene cloning methods cannot be used to genetically modify organisms.
4. Cross-pollination is considered to be a process through which plants can be …
  - genetically modified.
  - cloned.
  - hormone injected.
  - exactly replicated.
5. Which of the following can genetically modify plants or animals?
  - Farmers/gardeners
  - Scientists
  - Animals
  - All of the above
6. What will happen to the genetic offspring of plants and animals that have been genetically modified?
  - The genes will be passed to the new offspring.
  - The offspring’s genetic makeup will revert back to its original state.
  - A genetic mutation will occur.
  - They will be physically or mentally disabled.
7. Injecting hormones into a plant or animal may change what about that organism?
  - the size of the plant or animal
  - the genetic makeup of that plant or animal
  - all of the above
  - none of the above
8. Adding to or inhibiting a plant’s or animal’s DNA occurs only in …
  - laboratories
  - nature
  - farms
  - all of the above
9. When were processes used to modify a plant’s or animal’s DNA developed?
  - In the past 10 years
  - In the past 50 years
  - In the past 100 years
  - Longer than 100 years
10. Methods that are NOT used in producing genetically modified foods include which of the following?
  - Gene cloning methods
  - Hormone injection
  - Cross-pollination
  - Selective pollination

## Appendix C. Scientific Epistemic Beliefs Questionnaire

**Table A1:** Scientific epistemic beliefs questionnaire items.

Dimension	Description	Score
Source	Everybody has to believe what scientists say.	
	In science, you have to believe what the science books say about stuff.	
	Whatever the teacher says in science class is true.	
	If you read something in a science book, you can be sure it’s true.	
	Only scientists know for sure what is true in science.	
Certainty	All questions in science have one right answer.	
	The most important part of doing science is coming up with the right answer.	
	Scientists pretty much know everything about science; there is not much more to know.	
	Scientific knowledge is always true.	
	Once scientists have a result from an experiment, that is the only answer.	
	Scientists always agree about what is true in science.	
Development	Some ideas in science today are different than what scientists used to think.	
	The ideas in science books sometimes change.	
	There are some questions that even scientists cannot answer.	
	Ideas in science sometimes change.	
	New discoveries can change what scientists think is true.	
	Sometimes scientists change their minds about what is true in science	
Justification	Ideas about science experiments come from being curious and thinking about how things work.	
	In science, there can be more than one way for scientists to test their ideas.	
	One important part of science is doing experiments to come up with new ideas about how things work.	
	It is good to try experiments more than once to make sure of your findings.	
	Good ideas in science can come from anybody, not just from scientists.	
	A good way to know if something is true is to do an experiment.	
	Good answers are based on evidence from many different experiments.	
	Ideas in science can come from your own questions and experiments.	
	It is good to have an idea before you start an experiment.	

## Appendix D. Epistemic Emotions Questionnaire

**Table A2:** Epistemic emotions questionnaire.

Dimension	Not at All	Very Little	Moderate	Strong	Very Strong
Anger					
Hopeful					
Anxious					
Bored					
Curious					
Happy					
Fearful					
Confused					
Interested					
Surprised					
Frustrated					
Hopeless					
Enjoyment					

## Appendix E. Controlling for Total Reading Time

To assess whether the associations between the cognitive and affective variables and visual myside bias were sensitive to overall reading duration, a supplementary multiple linear regression analysis was conducted with revisited fixation count (RFC) difference as the dependent variable. Prior knowledge, scientific epistemic beliefs, negative epistemic emotions, and total reading time (TRT) were entered simultaneously as predictors. The analysis included the same 57 participants as the primary regression model reported in Table 7 of the main manuscript.

**Table A3:** Multiple Linear Regression Analysis Predicting RFC Difference After Controlling for Total Reading Time.

Predictor	B	SE	β	*p*	VIF
Constant	47.202	18.462	—	0.014	—
Prior knowledge	−1.471	0.441	−0.430	0.002	1.552
Scientific epistemic beliefs	−8.131	3.878	−0.256	0.041	1.387
Negative epistemic emotions	0.24	0.267	0.128	0.373	1.88
Total reading time	0.00084	0.01037	0.008	0.935	1.014

Note. RFC = revisited fixation count; = unstandardized regression coefficient; SE = standard error; β = standardized regression coefficient; VIF = variance inflation factor. RFC difference was calculated by subtracting the otherside AOI value from the corresponding myside AOI value. Total reading time was entered in seconds. The overall model was statistically significant, F(4, 52) = 10.338, *p* < 0.001, R^2^ = 0.443, adjusted R^2^ = 0.400. Compared with the primary model without TRT, the inclusion of TRT resulted in a negligible increase in explained variance (ΔR^2^ < 0.001). The change in model fit was not statistically significant, ΔF(1, 52) = 0.007, *p* = 0.935.

The standardized regression coefficients for prior knowledge (β = −0.430) and scientific epistemic beliefs (β = −0.256) remained similar to those obtained in the primary RFC difference model (β = −0.429 and β = −0.255, respectively). Negative epistemic emotions remained a nonsignificant predictor after TRT was included. Thus, the inclusion of overall reading duration did not materially change the estimated associations in the primary RFC difference model.

## Appendix F. Item-Level Statistics for the Prior Knowledge Test

**Table A4:** Item-Level Statistics for the Prior Knowledge Test.

Item	Difficulty Index	Item Variance	Corrected Item–Total Correlation	KR-20 if Item Delete
1	1.000	0.000	—	0.619
2	1.000	0.000	—	0.619
3	1.000	0.000	—	0.619
4	0.965	0.034	−0.112	0.645
5	0.912	0.081	0.452	0.545
6	0.807	0.159	0.497	0.517
7	0.579	0.248	0.610	0.461
8	0.246	0.189	0.535	0.499
9	0.123	0.110	0.365	0.563
10	0.035	0.034	−0.157	0.651

## Appendix G. Comparison of the Original and Reduced Prior Knowledge Scores

**Table A5:** Comparison of the Original and Reduced Prior Knowledge Scores.

Outcome	Original 10-Item Score	Reduced 8-Item Score
KR-20	0.603	0.662
Mean	6.67	5.67
SD	1.37	1.37
Range	4–9	3–8
Correlation with PTS difference	−0.615	−0.615
Correlation with RFC difference	−0.571	−0.571
Correlation with post-reading myside bias	−0.716	−0.716

Note. Because Items 4 and 10 contributed an almost constant combined score across participants, removal of these items shifted the total score while leaving the rank ordering unchanged. Identical correlations therefore reflect this score transformation and should not be interpreted as evidence of robustness to item removal.

## Appendix H. Myside Bias Indicators by Pre-Reading Stance Strength

**Table A6:** Myside Bias Indicators by Pre-Reading Stance Strength.

Stance Strength	*n*	PTS Difference, M (SD)	Range	RFC Difference, M (SD)	Range	Post-Reading Myside Bias, M (SD)	Range
Weak (3–4)	35	6.18 (3.95)	−2.08–13.66	9.34 (5.05)	1–20	1.11 (1.02)	0–4
Moderate (2–5)	19	6.76 (3.06)	0.59–12.75	9.11 (4.08)	2–17	1.00 (0.88)	0–3
Strong (1–6)	3	6.77 (3.48)	2.94–9.73	9.67 (4.16)	5–13	1.00 (1.00)	0–2
